# Supplementary material for: Stress granule assembly impairs macrophage efferocytosis to aggravate allergic rhinitis in mice
Source: Nat Commun. 2025 Jul 1;16:5610. doi: 10.1038/s41467-025-60920-0 (PMC12218239; doi:10.1038/s41467-025-60920-0)
Supplement: Supplementary file 1 — Supplementary information [file 41467_2025_60920_MOESM1_ESM.pdf]

Supplementary Information

Stress granule assembly impairs macrophage efferocytosis to aggravate allergic rhinitis in mice

Zhou *et al.*

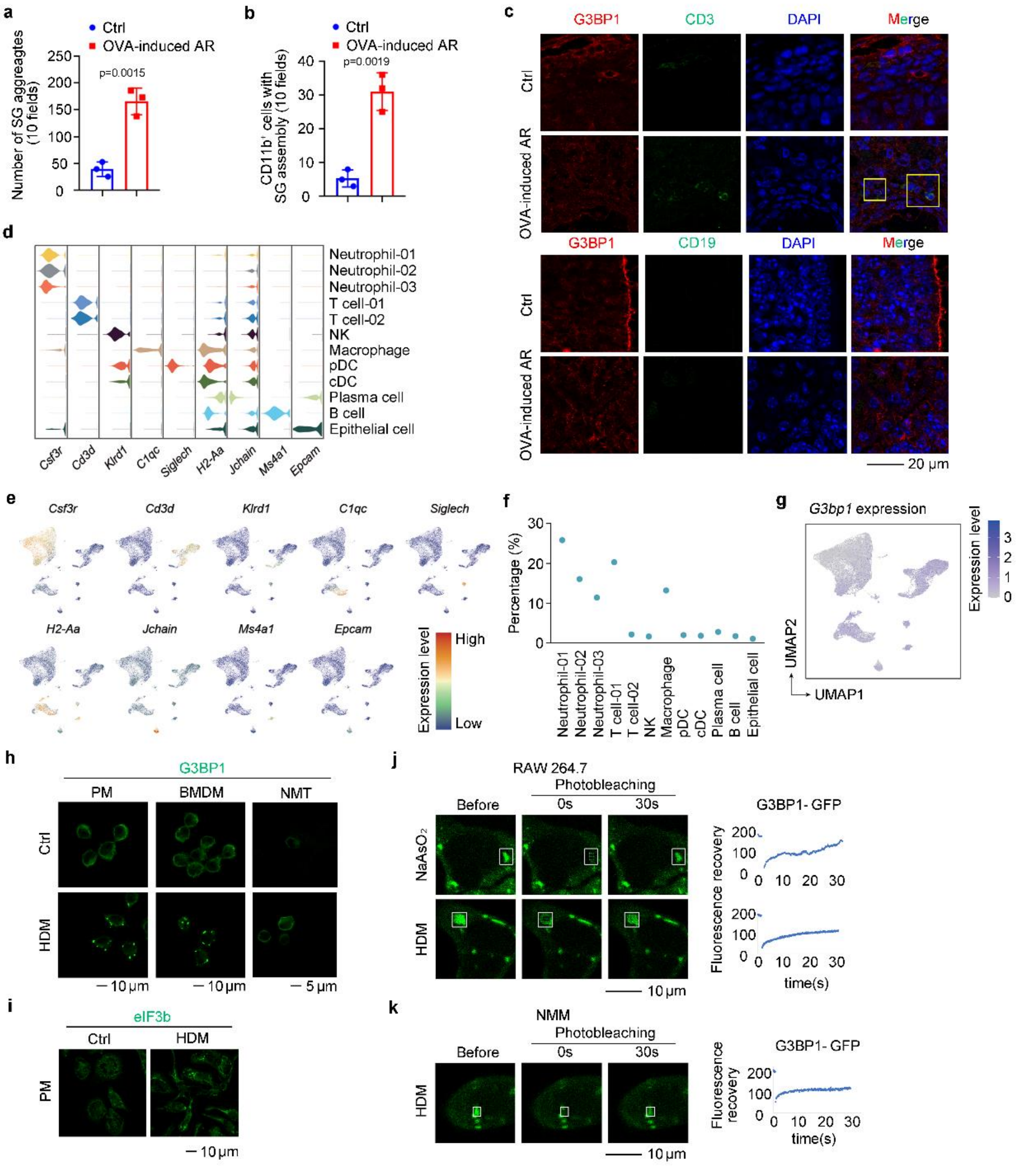

Supplementary Fig. 1 | SGs are assembled in the macrophages of nasal mucosa during AR

a, Quantification of G3BP1 aggregates per random 10 fields (n=3, biological replicates). (two-tailed unpaired Student’s t test).

b, Quantification of CD11b^{+} cells with G3BP1 aggregates per random 10 fields (n=3, biological replicates). (two-tailed unpaired Student’s t test).

c, Representative immunofluorescence assay showing G3BP1 and CD3 or CD19 in the nasal mucosa from control and AR mice.

d, Violin plots showing the expression levels of marker genes in the 12 cell clusters.

e, UMAP plots displaying the expression levels of marker genes.

f, The fraction of 12 cell clusters originating from the $CD45^+$ cells of nasal mucosa.

g, UMAP visualization of *G3bp1* expression in the $CD45^+$ cells from nasal mucosa.

h, Representative immunofluorescence assay showing G3BP1 in PMs, BMDMs, and NMTs under HDM (100 μg/well) stimulus.

i, Representative immunofluorescence assay showing eIF3b in PMs under HDM (100 μg/well) stimulus.

j, FRAP imaging of G3BP1-GFP in RAW 264.7 exposed to $NaAsO_2$ (50 μM) for 30 min or HDM (100 μg/well) for 1 h.

k, FRAP imaging of G3BP1-GFP in NMMs exposed to HDM (100 μg/well) for 1 h.

Data are shown as mean ± s.d. or photographs from one representative of three independent experiments or directly as indicated.

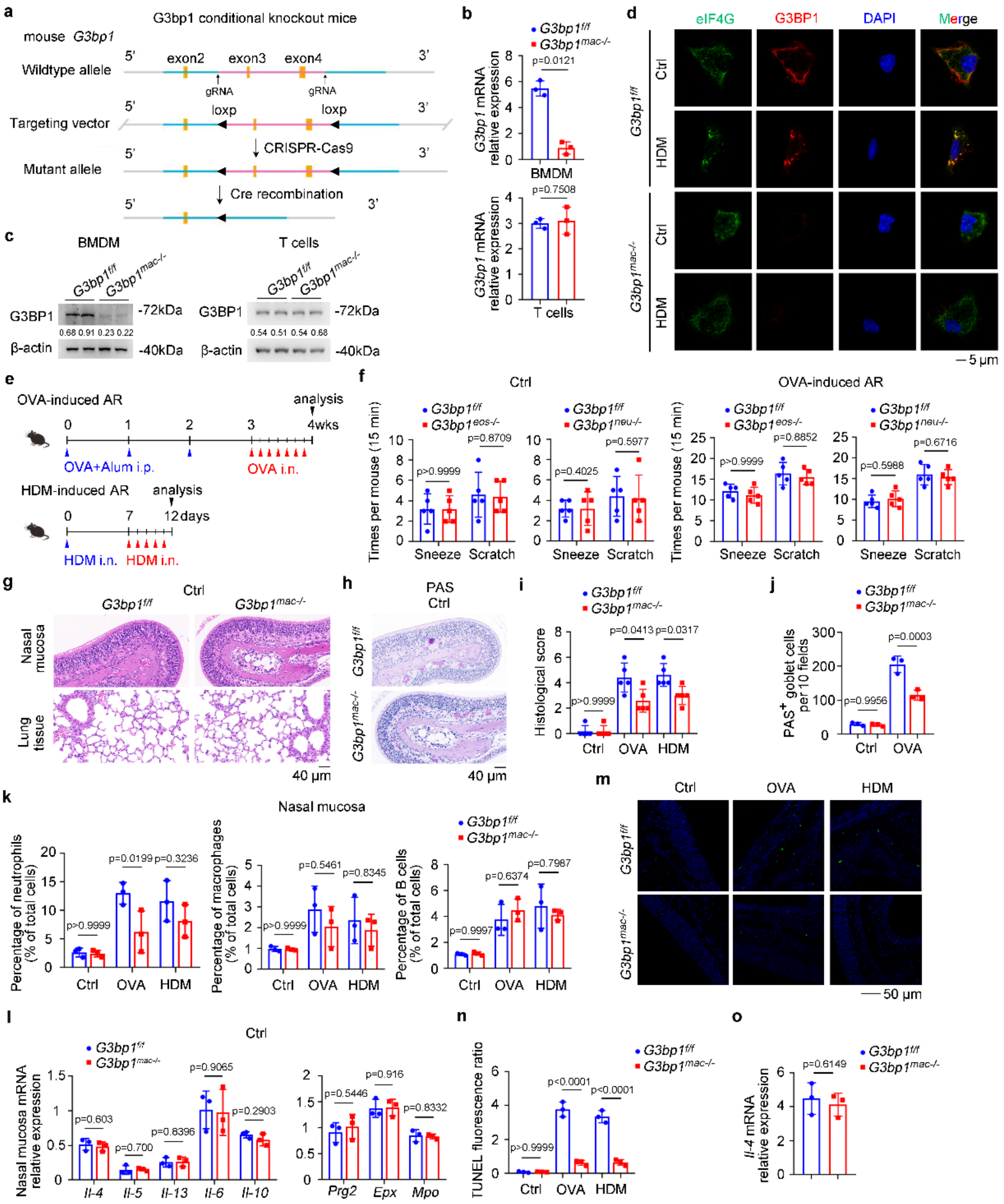

Supplementary Fig. 2 | SG assembly in macrophages promotes AR progression

a, Construction of *G3bp1* conditional knockout mice.

b, *G3bp1* mRNA expression in BMDMs and splenic T cells of *G3bp1*$^{f/f}$ and *G3bp1*$^{mac-/-}$ mice (n=3, biological replicates). (two-tailed unpaired Student's t test).

c, G3BP1 protein level in BMDMs and splenic T cells of *G3bp1*$^{f/f}$ and *G3bp1*$^{mac-/-}$ mice.

d, Representative immunofluorescence assay showing G3BP1 and eIF4G in BMDMs of *G3bp1*$^{f/f}$ and *G3bp1*$^{mac-/-}$ mice under HDM (100 μg/well) stimulus.

e, Design of AR mouse models.

f, *G3bp1*$^{f/f}$, *G3bp1*$^{eos-/-}$, and *G3bp1*$^{neu-/-}$ mice were administrated with OVA to induce AR symptoms. Times of sneezes and scratches in each mouse was counted in 15 min after last i.n. challenge (n=5, biological replicates). (two-tailed unpaired Student's t test).

g,h, H&E and PAS staining of the nasal mucosa and lung tissues from *G3bp1*$^{f/f}$ and *G3bp1*$^{mac-/-}$ control mice.

i, Assessment of histological damage using histological damage scoring in control, OVA and HDM group (n=5, biological replicates). (Mann-Whitney U test).

j, Assessment of PAS$^+$ goblet cells in nasal mucosa from *G3bp1*$^{f/f}$ and *G3bp1*$^{mac-/-}$ mice per 10 random fields using ImageJ software (n=3, biological replicates). (Tukey's HSD).

k, Flow cytometry of neutrophils, macrophages, and B cells in the nasal mucosa of control and AR mice (n=3, biological replicates). (Tukey's HSD).

l, mRNA expressions of cytokines, *Prg2*, *Epx*, and *Mpo* were detected by qRT-PCR in the nasal mucosa of *G3bp1*$^{f/f}$ and *G3bp1*$^{mac-/-}$ control mice (n=3, biological replicates). (two-tailed unpaired Student's t test).

m, Representative TUNEL staining showing apoptotic cells in the nasal mucosa of control and AR mice.

n, Assessment of TUNEL staining fluorescence ratio in nasal mucosa from *G3bp1*$^{f/f}$ and *G3bp1*$^{mac-/-}$ mice per 10 random fields using ImageJ software (n=3, biological replicates). (Tukey's HSD).

o, mRNA expressions of *Il-4* were detected by qRT-PCR in Th2 cells of *G3bp1*$^{f/f}$ and *G3bp1*$^{mac-/-}$ mice (n=3, biological replicates). (two-tailed unpaired Student's t test).

Data are shown as mean ± s.d. or photographs from one representative of three independent experiments.

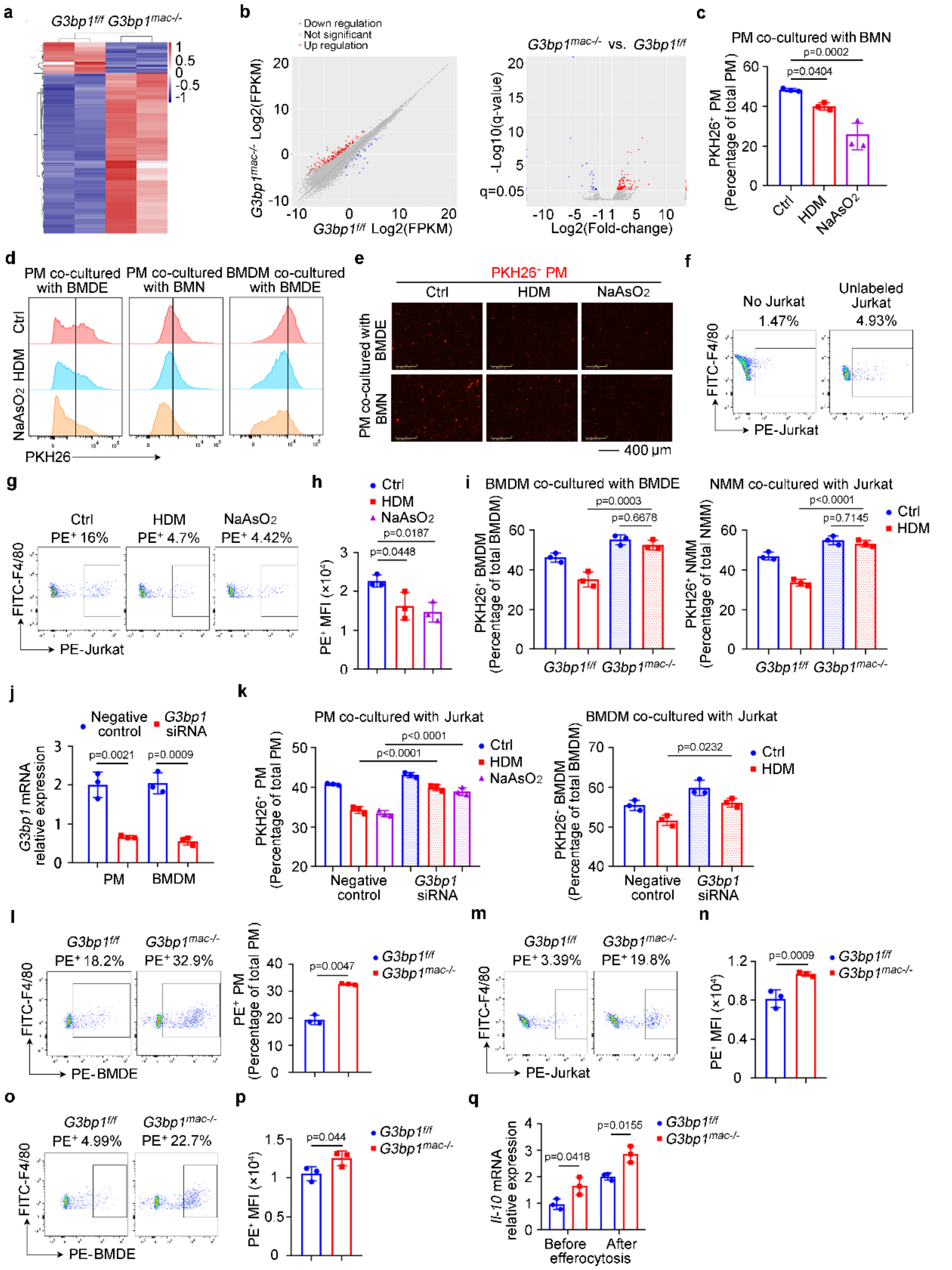

Supplementary Fig. 3 | SG assembly in macrophages impairs efferocytosis

a, Heatmap showing the differentially expressed genes between *G3bp1*$^{f/f}$ and *G3bp1*$^{mac-/-}$ macrophages from RNA-seq data.

b, Scatter dot plot and volcano plot showing the genes differentially expressed between *G3bp1*$^{f/f}$ and *G3bp1*$^{mac-/-}$ macrophages from RNA-seq data.

c, HDM (100 μg/well) or $NaAsO_2$ (50 μM)-inhibited in vitro efferocytosis of apoptotic cells (labelled with PKH26) by macrophages was measured by flow cytometry (n=3, biological replicates). (Dunnett's multiple comparisons test).

d, Representative flow cytometry showing $PKH26^+$ macrophages (engulfment of apoptotic cells) administrated with HDM (100 μg/well) or $NaAsO_2$ (50 μM) in the efferocytosis assay.

e, Incucyte imaging showing $PKH26^+$ macrophages (engulfment of apoptotic cells) administrated with HDM (100 μg/well) or $NaAsO_2$ (50 μM) in the efferocytosis assay.

f, Representative flow cytometry showing no-Jurkat control and unlabeled Jurkat control in the efferocytosis assay related to Fig. 3f.

g, Representative flow cytometry showing PE^{high} frequency by adjusting the PE^+ gate to 5k on the PE-Jurkat axis related to Fig. 3f.

h, MFI value of PE^+ fraction (gate to 5k) in **g** (n=3, biological replicates). (Dunnett's multiple comparisons test).

i, In vitro efferocytosis assay of PKH26-labelled apoptotic BMDEs or Jurkat cells engulfed by $G3bp1^{f/f}$ and $G3bp1^{mac-/-}$ macrophages was measured by flow cytometry (n=3, biological replicates). (Tukey's HSD).

j, Knockdown efficiency of *G3bp1* was examined by qRT-PCR (n=3, biological replicates). (two-tailed unpaired Student's t test).

k, In vitro efferocytosis assay of PKH26-labelled apoptotic Jurkat cells engulfed by *G3bp1* knockdown macrophages was measured by flow cytometry (n=3, biological replicates). (Tukey's HSD).

l, In vivo efferocytosis assay of PKH26-labelled apoptotic BMDEs engulfed by $G3bp1^{f/f}$ and $G3bp1^{mac-/-}$ PMs was measured by flow cytometry (n=3, biological replicates). (two-tailed unpaired Student's t test with Welch's correction).

m, Representative flow cytometry showing PE^{high} frequency by adjusting the PE^+ gate to 5k on the PE-Jurkat axis related to Fig. 3j.

n, MFI value of PE^+ fraction (gate to 5k) in **m** (n=3, biological replicates). (two-tailed unpaired Student's t test).

o, Representative flow cytometry showing PE^{high} frequency by adjusting the PE^+ gate to 5k on the PE-Jurkat axis related to **l**.

p, MFI value of PE^+ fraction (gate to 5k) in **o** (n=3, biological replicates). (two-tailed unpaired Student's t test).

q, mRNA expressions of *Il-10* were detected by qRT-PCR in the *G3bp1*$^{f/f}$ and *G3bp1*$^{mac-/-}$ PMs co-cultured with or without apoptotic Jurkat cells (n=3, biological replicates). (Tukey's HSD).

Data are shown as mean ± s.d. or photographs from one representative of three independent experiments.

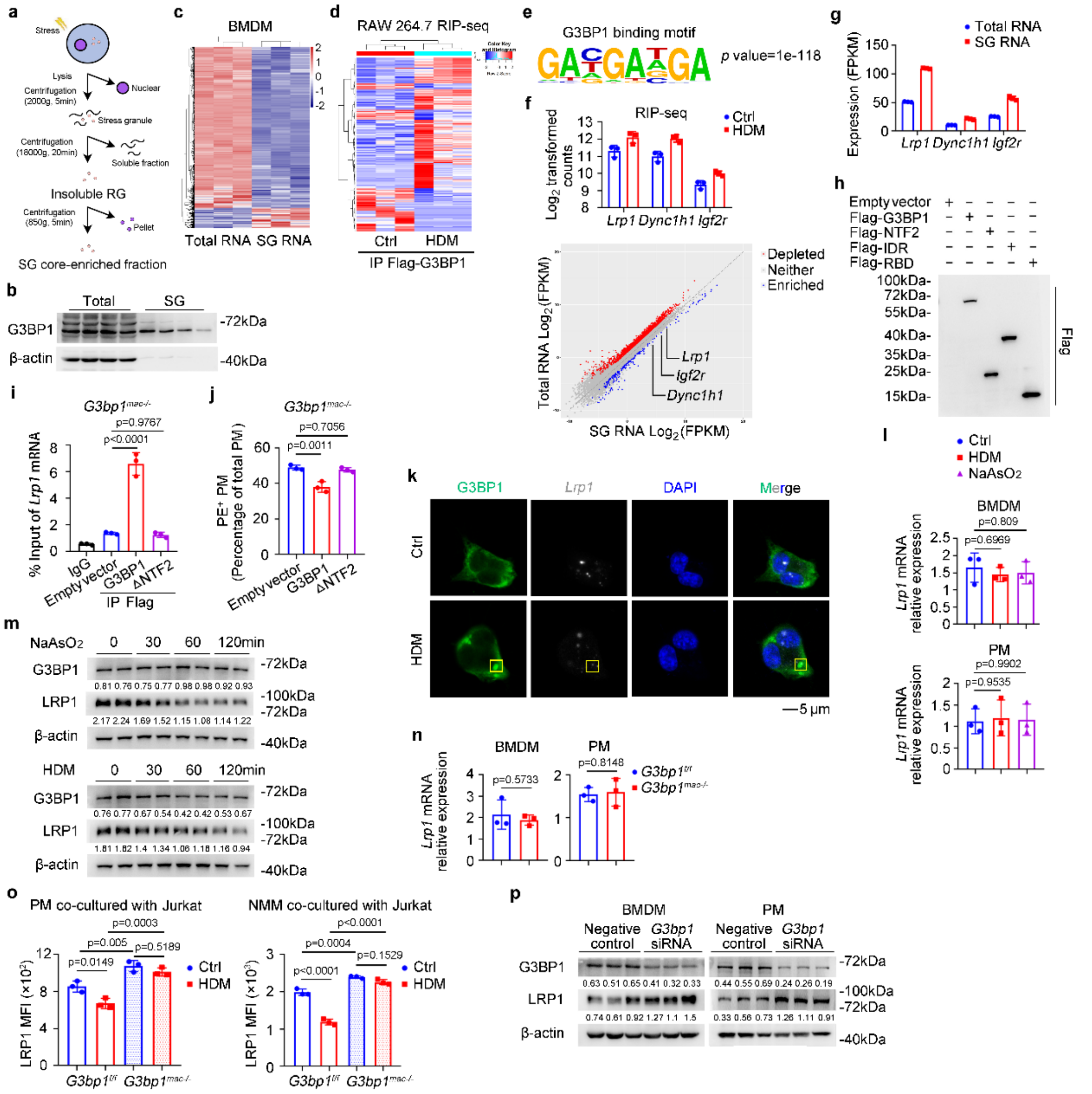

Supplementary Fig. 4 | SG assembly in macrophages sequesters *Lrp1* mRNA to suppress its translation

a, Workflow of SG isolation.

b, Confirmation of total lysate and SG core-enriched fraction.

c, Heatmap showing the differentially expressed genes between total lysate and SG sore-enriched fraction of BMDMs from SG RNA-seq data.

d, Heatmap showing the differential genes of RAW 264.7 cells overexpressed with Flag-tagged G3BP1 between control and HDM administration from RIP-seq data.

e, Top consensus motif identified by HOMER with RIP-seq peaks.

f, Levels of G3BP1-binding mRNAs (*Lrp1*, *Dync1h1*, *Igf2r*) from RIP-seq data.

g, Levels of SG core-enriched mRNAs (*Lrp1*, *Dync1h1*, *Igf2r*) from SG RNA-seq data. Scatter dot plot displaying the enrichment or depletion of SG-purified mRNAs

compared with total mRNAs.

h, Flag-tagged G3BP1 truncates, including NTF2 domain, intrinsically disordered region (IDR), and RNA binding domain (RBD), were constructed.

i, The association between G3BP1 and *Lrp1* mRNA in *G3bp1*$^{mac-/-}$ macrophages transfected with G3BP1 and ΔNTF2 (NTF2 domain deleted) was determined by RIP-qRT-PCR (n=3, biological replicates). (Tukey's HSD).

j, In vitro macrophage efferocytosis of apoptotic cells (labelled with PKH26) by *G3bp1*$^{mac-/-}$ macrophages transfected with G3BP1 and ΔNTF2 was measured by flow cytometry (n=3, biological replicates). (Dunnett's multiple comparisons test).

k, Immunofluorescence of G3BP1 and FISH imaging of *Lrp1* mRNA in PMs.

l, *Lrp1* mRNA expression was detected by qRT-PCR in macrophages administrated with HDM (100 μg/well) or $NaAsO_2$ (50 μM) (n=3, biological replicates). (Dunnett's multiple comparisons test).

m, G3BP1 and LRP1 protein levels in PMs administrated with HDM (100 μg/well) or $NaAsO_2$ (50 μM) were examined by Western blot.

n, *Lrp1* mRNA expression was detected by qRT-PCR in macrophages of *G3bp1*$^{f/f}$ and *G3bp1*$^{mac-/-}$ mice (n=3, biological replicates). (two-tailed unpaired Student's t test).

o, LRP1 protein level on PMs and NMMs of *G3bp1*$^{f/f}$ and *G3bp1*$^{mac-/-}$ mice was examined by flow cytometry in the in vitro efferocytosis assay (n=3, biological replicates). (Tukey's HSD).

p, G3BP1 and LRP1 protein levels in primary macrophages with *G3bp1* knockdown were examined by Western blot.

Data are shown as mean ± s.d. or photographs from one representative of three independent experiments.

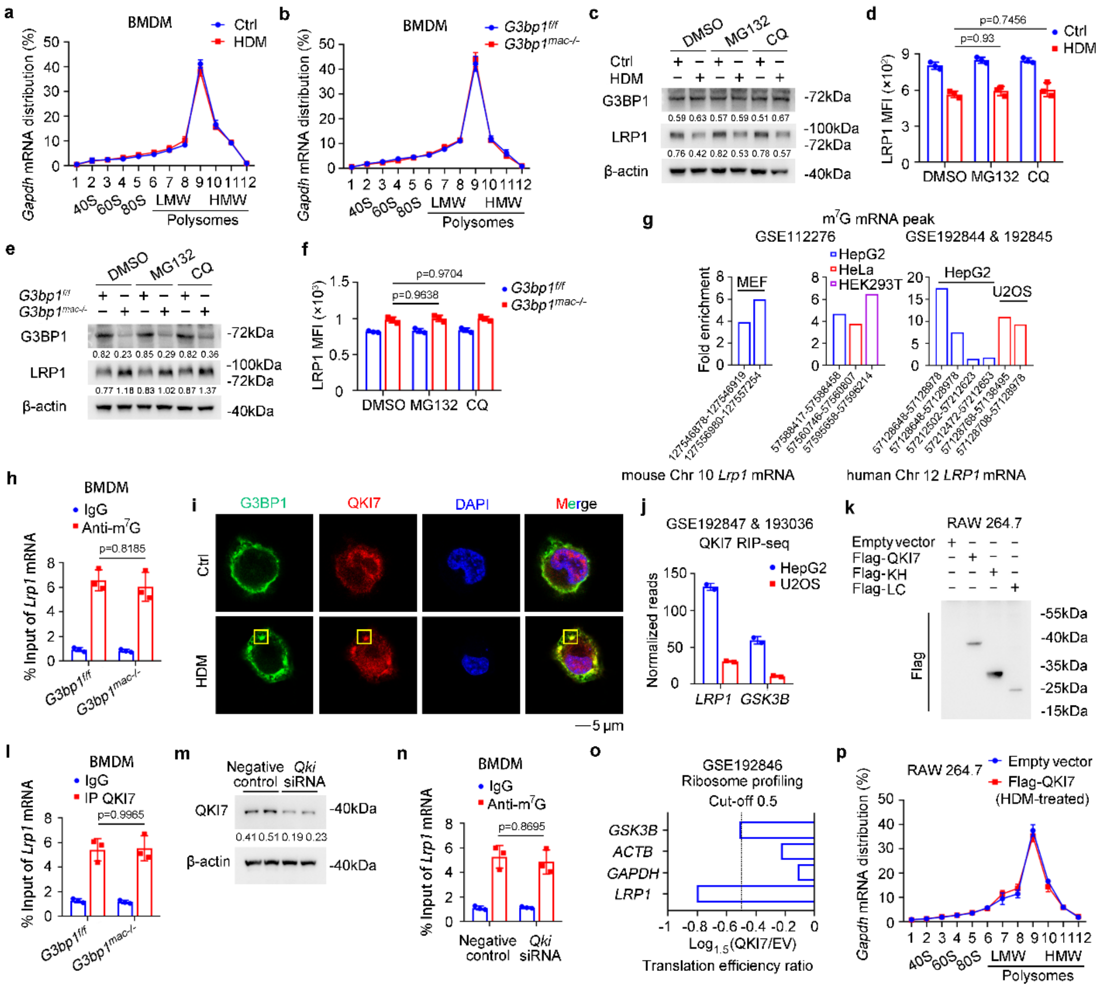

Supplementary Fig. 5 | Internal m^{7}G-modified *Lrp1* mRNA is shuttled by QKI7 into SGs thus repressing its translation

a, BMDMs were administrated with HDM (200 μg/well), and relative *Gapdh* mRNA distribution in each ribosome fractions was analyzed by qRT-PCR (n=3, biological replicates). (Tukey's HSD).

b, In BMDMs from *G3bp1$^{f/f}$* and *G3bp1$^{mac-/-}$* mice, relative *Gapdh* mRNA distribution in each ribosome fractions was analyzed by qRT-PCR (n=3, biological replicates). (Tukey's HSD).

c, G3BP1 and LRP1 protein levels in PMs administrated with HDM combining MG132 or CQ were examined by Western blot.

d, LRP1 protein levels in PMs administrated with HDM combining MG132 or CQ were examined by flow cytometry (n=3, biological replicates). (Tukey's HSD).

e, G3BP1 and LRP1 protein levels in PMs of *G3bp1$^{f/f}$* and *G3bp1$^{mac-/-}$* mice administrated with MG132 or CQ were examined by Western blot.

f, LRP1 protein levels in PMs of *G3bp1*$^{f/f}$ and *G3bp1*$^{mac-/-}$ mice administrated with MG132 or CQ were examined by flow cytometry (n=3, biological replicates). (Tukey's HSD).

g, MeRIP-seq (GSE112276, 192844 & 192845) showing the enrichment of internal m^{7}G modification in *Lrp1* mRNA of MEF and *LRP1* mRNA of HepG2, HeLa, HEK293T, and U2OS cells as indicated.

h, m^{7}G modification of *Lrp1* mRNA in BMDMs of *G3bp1*$^{f/f}$ and *G3bp1*$^{mac-/-}$ mice was examined by m^{7}G-RIP-qRT-PCR (n=3, biological replicates). (Tukey's HSD).

i, Representative immunofluorescence imaging of G3BP1 and QKI7 in PMs under HDM (100 μg/well).

j, RIP-seq (GSE192847 & 193036) showing the association of QKI7 with *LRP1* and *GSK3B* mRNAs in HepG2 and U2OS cells.

k, Flag-tagged QKI7 truncates, including KH domain and LC domain, were constructed.

l, The association between QKI7 and *Lrp1* mRNA in BMDMs of *G3bp1*$^{f/f}$ and *G3bp1*$^{mac-/-}$ mice was examined by RIP-qRT-PCR (n=3, biological replicates). (Tukey's HSD).

m, QKI7 knockdown efficiency was examined by Western blot.

n, m^{7}G modification of *Lrp1* mRNA in BMDMs with QKI7 knockdown was examined by m^{7}G-RIP-qRT-PCR (n=3, biological replicates). (Tukey's HSD).

o, Ribo-seq (GSE192846) showing the translational efficiency of the indicated mRNAs in empty vector (EV) or QKI7 overexpressed U2OS cells under $NaAsO_2$ administration. ACTB and GAPDH were used as the negative controls.

p, In QKI7-overexpressed RAW 264.7 cells administrated with HDM (200 μg/well), relative *Gapdh* mRNA distribution in each ribosome fractions was analyzed by qRT-PCR (n=3, biological replicates). (Tukey's HSD).

Data are shown as mean ± s.d. or photographs from one representative of three independent experiments.

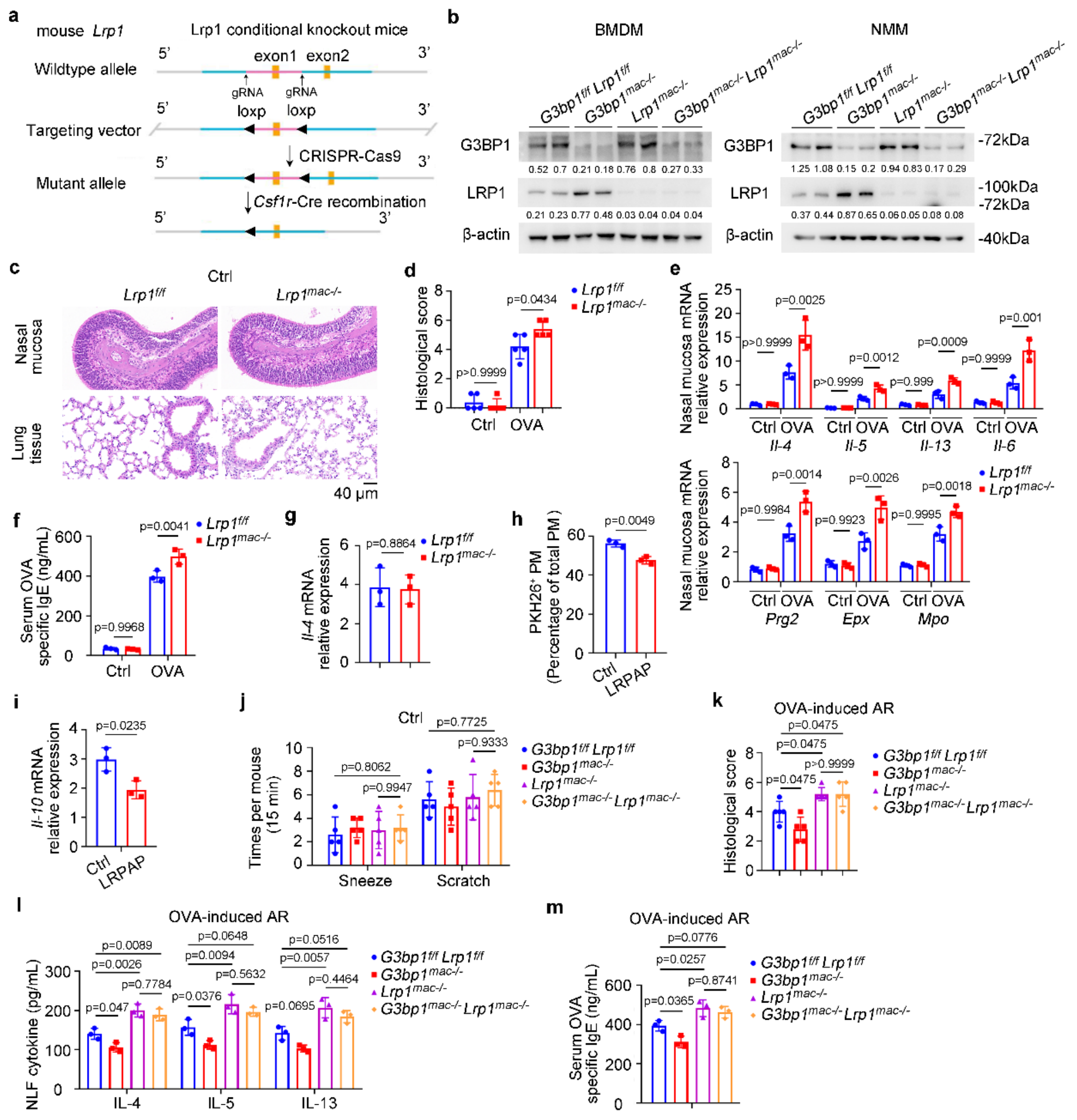

Supplementary Fig. 6 | The SG-promoted AR depends on the inhibition of efferocytosis receptor LRP1

a, Construction of macrophage-specific *Lrp1* knockout mice.

b, G3BP1 and LRP1 protein levels in PMs and NMMs of *G3bp1*$^{f/f}$*Lrp1*$^{f/f}$, *G3bp1*$^{mac-/-}$, *Lrp1*$^{mac-/-}$, and *G3bp1*$^{mac-/-}$*Lrp1*$^{mac-/-}$ mice were examined by Western blot.

c, H&E staining of the nasal mucosa and lung tissues from *Lrp1*$^{f/f}$ and *Lrp1*$^{mac-/-}$ control mice.

d, Assessment of histological damage using histological damage scoring in control and OVA group (n=5, biological replicates). (Mann-Whitney U test).

e, mRNA expressions of cytokines, *Prg2*, *Epx*, and *Mpo* were detected by qRT-PCR analysis in the nasal mucosa of *Lrp1*$^{f/f}$ and *Lrp1*$^{mac-/-}$ mice (n=3, biological replicates). (Tukey's HSD).

f, Serum OVA-specific IgE was detected by ELISA in *Lrp1*$^{f/f}$ and *Lrp1*$^{mac-/-}$ mice (n=3, biological replicates). (Tukey's HSD).

g, mRNA expressions of *Il-4* were detected by qRT-PCR in Th2 cells of *Lrp1*$^{f/f}$ and *Lrp1*$^{mac-/-}$ mice (n=3, biological replicates). (two-tailed unpaired Student's t test).

h, LRPAP (500 nM) treatment-inhibited in vitro macrophage efferocytosis of apoptotic cells (labelled with PKH26) was measured by flow cytometry (n=3, biological replicates). (two-tailed unpaired Student's t test).

i, mRNA expression of *Il-10* in macrophages pretreated with LRPAP was detected by qRT-PCR analysis after in vitro efferocytosis assay (n=3, biological replicates). (two-tailed unpaired Student's t test).

j, Times of sneezes and scratches in each control mouse was counted in 15 min (n=5, biological replicates). (Dunnett's multiple comparisons test).

k, Assessment of histological damage using histological damage scoring in OVA group (n=5, biological replicates). (Dunnett's multiple comparisons test).

l, IL-4, IL-5, and IL-13 productions in NLF were detected by ELISA in *G3bp1*$^{f/f}$*Lrp1*$^{f/f}$, *G3bp1*$^{mac-/-}$, *Lrp1*$^{mac-/-}$, and *G3bp1*$^{mac-/-}$*Lrp1*$^{mac-/-}$ AR mice (n=3, biological replicates). (Dunnett's multiple comparisons test).

m, Serum OVA-specific IgE was detected by ELISA in *G3bp1*$^{f/f}$*Lrp1*$^{f/f}$, *G3bp1*$^{mac-/-}$, *Lrp1*$^{mac-/-}$, and *G3bp1*$^{mac-/-}$*Lrp1*$^{mac-/-}$ AR mice (n=3, biological replicates). (Dunnett's multiple comparisons test).

Data are shown as mean ± s.d. or photographs from one representative of three independent experiments.

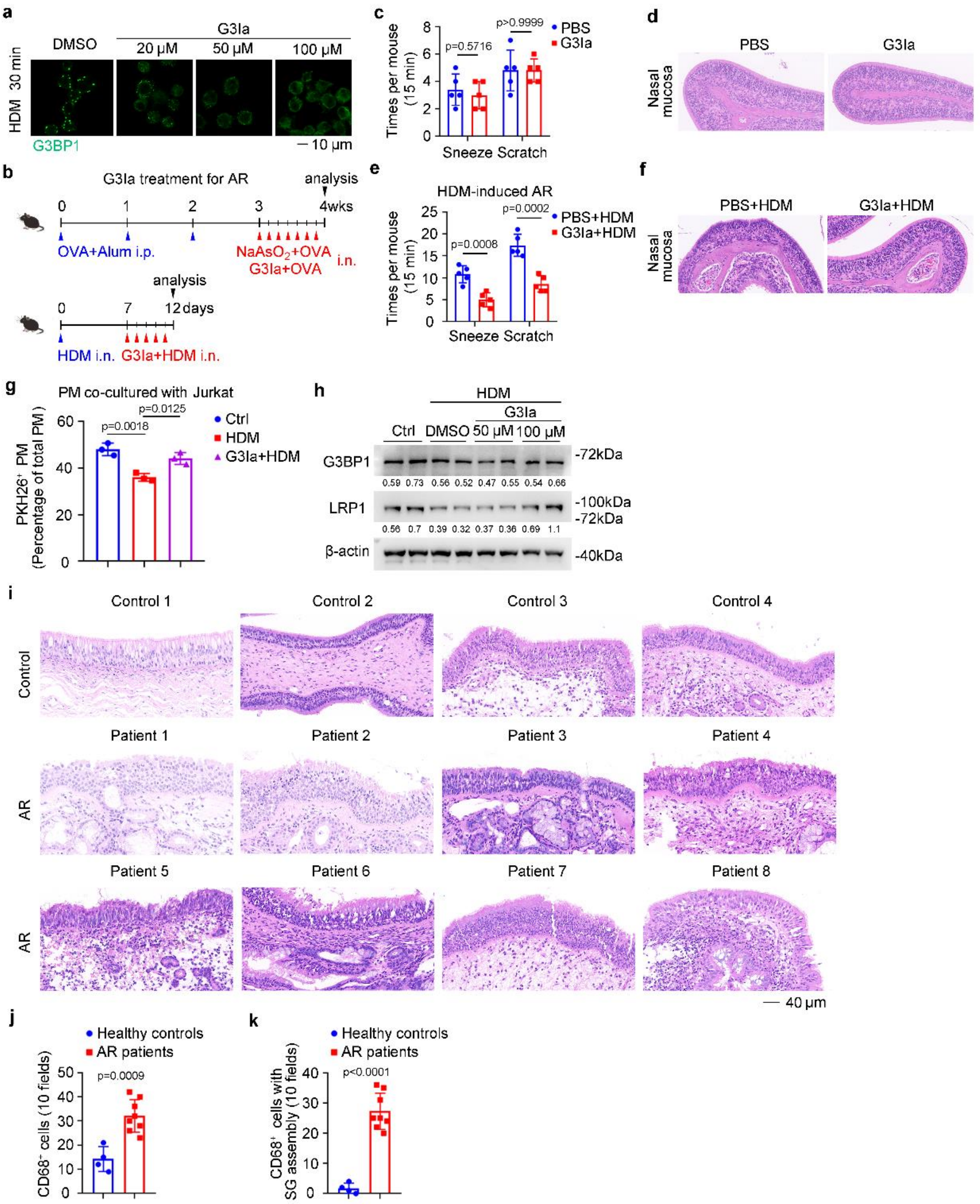

Supplementary Fig. 7 | SG assembly inhibitor alleviates AR symptoms, and SGs are assembled in nasal macrophages of AR patients

a, Representative immunofluorescence imaging of SG assembly (G3BP1) in BMDMs pre-treated with G3Ia for 20 min followed by HDM stimulation for 30 min.

b, Design of AR mouse model with SG assembly inhibitor treatment.

c, Times of sneezes and scratches in each mouse treated with PBS or G3Ia alone were counted in 15 min (n=5, biological replicates). (two-tailed unpaired Student's t test).

d, H&E staining of the nasal mucosa from mice treated with PBS or G3Ia alone.

e, Times of sneezes and scratches in each HDM-induced AR mouse were counted in 15 min (n=5, biological replicates). (two-tailed unpaired Student's t test).

f, H&E staining of the nasal mucosa from HDM-induced AR mice.

g, G3Ia-rescued macrophage efferocytosis of apoptotic Jurkat cells (labelled with PKH26) inhibited by HDM was measured by flow cytometry (n=3, biological replicates). (Dunnett's multiple comparisons test).

h, G3BP1 and LRP1 protein levels in PMs pre-treated with G3Ia for 20 min followed by HDM stimulation for 1 h were examined by Western blot.

i, H&E staining showing the nasal mucosa from healthy controls and AR patients.

j, Quantification of $CD68^+$ cells per random 10 fields (healthy control n=4, AR patients n=8, biological replicates). (two-tailed unpaired Student's t test).

k, Quantification of $CD68^+$ cells with G3BP1 aggregates per random 10 fields (healthy control n=4, AR patients n=8, biological replicates). (two-tailed unpaired Student's t test).

Data are shown as mean ± s.d. or photographs from one representative of three independent experiments.

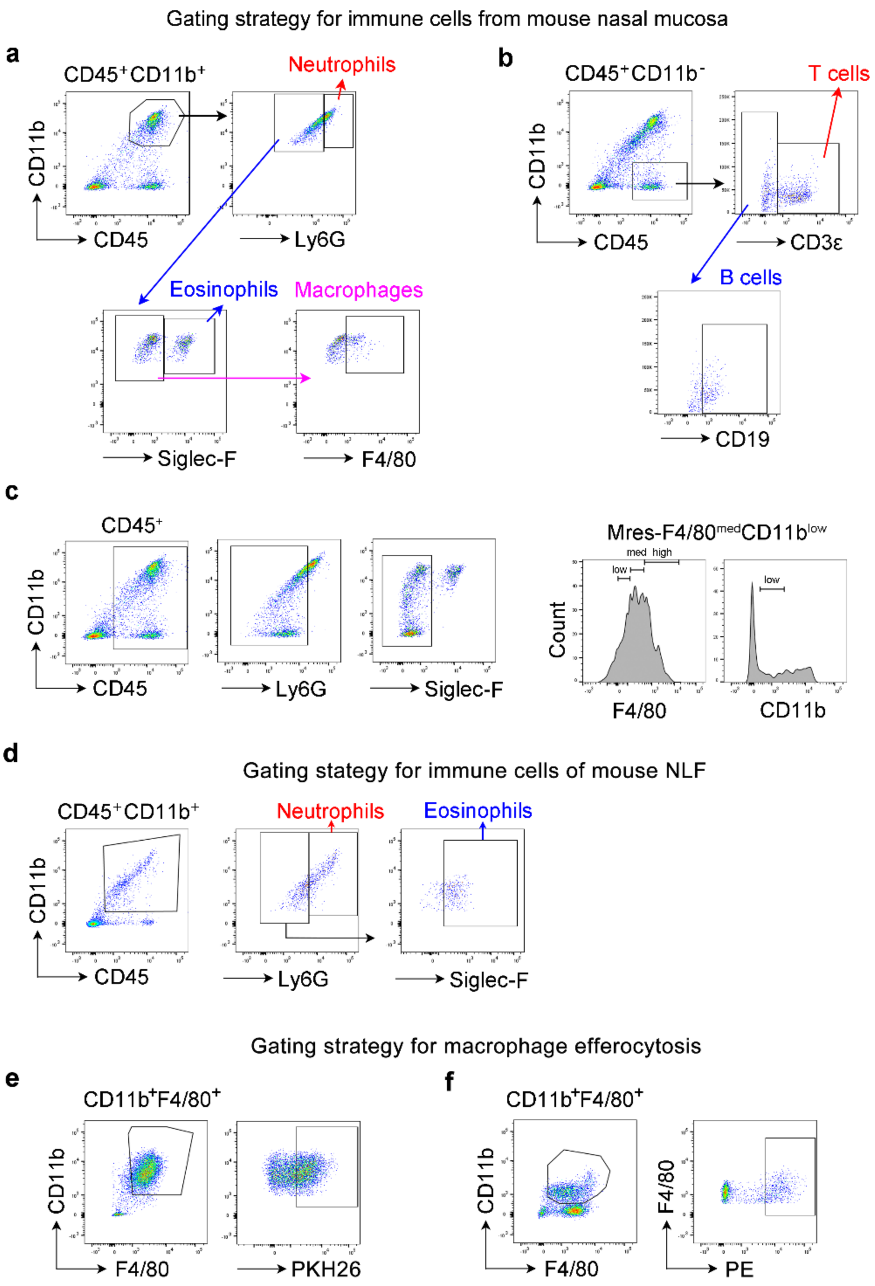

Supplementary Fig. 8 | Examples of flow cytometry gating.

a-c, Representative flow cytometry plots illustrating the gating strategy of immune cells in the nasal mucosa. **a,** Neutrophil, eosinophil, and macrophage gating (corresponded to Fig. 2d, Supplementary Fig. 2k, Fig. 6c,j, and Fig. 7c). **b,** T cell and B cell gating (corresponded to Fig. 2d, Supplementary Fig. 2k, Fig. 6c, and Fig. 7c). **c,** Mres ($F4/80^{med}CD11b^{low}$) gating (corresponded to Fig. 2f).

d, Representative flow cytometry plots illustrating the gating strategy of neutrophils and eosinophils in NLF (corresponded to Fig. 2e).

e-f, Representative flow cytometry plots illustrating the gating strategy of in vitro and in vivo efferocytosis of macrophages. **e,** in vitro efferocytosis (corresponded to Fig. 3c,d,g, Supplementary Fig. 3c,i,k, Fig. 6e, Supplementary Fig. 6h, and Supplementary Fig. 7g). **f,** in vivo efferocytosis (corresponded to Fig. 3f,j, Supplementary Fig. 3f,g,l,m,o, and Fig. 6g,k).
