# Supplementary material for: Stress granule assembly impairs macrophage efferocytosis to aggravate allergic rhinitis in mice
Source: Nat Commun. 2025 Jul 1;16:5610. doi: 10.1038/s41467-025-60920-0 (PMC12218239; doi:10.1038/s41467-025-60920-0)
Supplement: Supplementary file 3 — Reporting Summary [file 41467_2025_60920_MOESM3_ESM.pdf]

nature portfolio

Corresponding author(s): Jin Hou

Last updated by author(s): May 8, 2025

Reporting Summary

Nature Portfolio wishes to improve the reproducibility of the work that we publish. This form provides structure for consistency and transparency in reporting. For further information on Nature Portfolio policies, see our Editorial Policies and the Editorial Policy Checklist.

Statistics

For all statistical analyses, confirm that the following items are present in the figure legend, table legend, main text, or Methods section.

n/a	Confirmed	
☐	☒	The exact sample size (*n*) for each experimental group/condition, given as a discrete number and unit of measurement
☐	☒	A statement on whether measurements were taken from distinct samples or whether the same sample was measured repeatedly
☐	☒	The statistical test(s) used AND whether they are one- or two-sided<br>*Only common tests should be described solely by name; describe more complex techniques in the Methods section.*
☒	☐	A description of all covariates tested
☐	☒	A description of any assumptions or corrections, such as tests of normality and adjustment for multiple comparisons
☐	☒	A full description of the statistical parameters including central tendency (e.g. means) or other basic estimates (e.g. regression coefficient) AND variation (e.g. standard deviation) or associated estimates of uncertainty (e.g. confidence intervals)
☐	☒	For null hypothesis testing, the test statistic (e.g. *F*, *t*, *r*) with confidence intervals, effect sizes, degrees of freedom and *P* value noted<br>*Give P values as exact values whenever suitable.*
☒	☐	For Bayesian analysis, information on the choice of priors and Markov chain Monte Carlo settings
☒	☐	For hierarchical and complex designs, identification of the appropriate level for tests and full reporting of outcomes
☒	☐	Estimates of effect sizes (e.g. Cohen's *d*, Pearson's *r*), indicating how they were calculated

Our web collection on statistics for biologists contains articles on many of the points above.

Software and code

Policy information about availability of computer code

Data collection	Leica Confocal Microscope MDI8 (Leica) for immunofluorescence assay, FRAP assay and live-cell imaging. Tanon 4600SF (Tanon) for WB scan. Fortessa Flow Cytometer (BD) with FlowJo software (BD) for flow cytometry data acquisition. Quantitative real-time PCR (qPCR) was performed on the LightCycler (Roche). Illumina NovaSeq 6000 instrument (Illumina) was used for RNA-seq, RIP-seq and mRNA m7G MeRIP-seq. 10X Genomics Chromium Controller Instrument (10X Genomics) was used for scRNA-seq.
Data analysis	FlowJo version (10.8.1) for analysis of flow cytometry. GraphPad Prism (version 8.0) for biostatistical analysis and scientific graphing. Hisat2 (version 2.0.4) + Stringtie(version 1.3.0) for RNA-seq data analysis. Hisat2 (version 2.0.4) + HTSeq software (version 0.9.1) + edgeR for RIP-seq data analysis. Hisat2 (version 2.0.4) + MACS (version 1.4.2) + diffReps (version 1.55.6) for mRNA m7G MeRIP-seq data analysis.

For manuscripts utilizing custom algorithms or software that are central to the research but not yet described in published literature, software must be made available to editors and reviewers. We strongly encourage code deposition in a community repository (e.g. GitHub). See the Nature Portfolio guidelines for submitting code & software for further information.

Data

Policy information about availability of data

All manuscripts must include a data availability statement. This statement should provide the following information, where applicable:

- Accession codes, unique identifiers, or web links for publicly available datasets
- A description of any restrictions on data availability
- For clinical datasets or third party data, please ensure that the statement adheres to our policy

scRNA-seq data have been deposited in the GEO database under accession code GSE266238. RNA-seq, SG RNA-seq, G3BP1 RIP-seq and m7G MeRIP-seq data have been deposited in GEO database under accession codes GSE264134, GSE263848, GSE264137, and GSE263851 respectively. All the original unprocessed gels and images, and all the original source data of figures have been deposited and available at the public research database Mendeley Data Reserved https://data.mendeley.com/datasets/kxksmyc89v/5 and provided as Source Data file.

Research involving human participants, their data, or biological material

Policy information about studies with human participants or human data. See also policy information about sex, gender (identity/presentation), and sexual orientation and race, ethnicity and racism.

Reporting on sex and gender	No sex and gender-based analysis was performed, nor did sex or gender factor into choosing of this study.
Reporting on race, ethnicity, or other socially relevant groupings	No race, ethnicity, or other socially relevant factors are involved in this study.
Population characteristics	This study did not involve human research participants. Only human biospecimens were used in the study. Human nasal mucosa samples were obtained from patients during nasal surgery in Second Military Medical University (Shanghai, China), including deviated nasal septum without AR (as healthy controls), and AR patients with the diagnosis based on the symptoms and allergen-specific IgE level class ≥ 2.
Recruitment	Clinical data and samples were retrospectively obtained from Changzhen Hospital affiliated to Second Military Medical University, Shanghai, China. There was no relevant bias on the inclusion of these patients that could affect the further analyses.
Ethics oversight	This study was approved by the Committee on Ethics of Medicine, Second Military Medical University (2024SL128). All the tissue samples in this study were collected with written informed consent from the patients.

Note that full information on the approval of the study protocol must also be provided in the manuscript.

Field-specific reporting

Please select the one below that is the best fit for your research. If you are not sure, read the appropriate sections before making your selection.

☒ Life sciences ☐ Behavioural & social sciences ☐ Ecological, evolutionary & environmental sciences

For a reference copy of the document with all sections, see nature.com/documents/nr-reporting-summary-flat.pdf

Life sciences study design

All studies must disclose on these points even when the disclosure is negative.

Sample size	No statistical method was used to determine sample size, but our sample sizes are similar to those reported in previous publication (Wen Chen, et al. Nature Immunology, 2022)
Data exclusions	No data were excluded from the analysis.
Replication	All experimental replicates are described in the figure legends.
Randomization	Mice were randomly assigned to experimental vs. control groups for all in vivo experiments.
Blinding	The H&E and PAS staining was performed by researchers blinded for allocation, and the measurement of AR symptoms was done by researchers blinded for allocation. The investigators were blinded to samples at the time of performing flow cytometry, real-time qRT-PCR, ELISA. For Western blotting, blinding was not performed. The investigators were blinded during data collection, and not blinded during data analysis.

Reporting for specific materials, systems and methods

We require information from authors about some types of materials, experimental systems and methods used in many studies. Here, indicate whether each material, system or method listed is relevant to your study. If you are not sure if a list item applies to your research, read the appropriate section before selecting a response.

Materials & experimental systems

n/a	Involved in the study
☐	☒ Antibodies
☐	☒ Eukaryotic cell lines
☒	☐ Palaeontology and archaeology
☐	☒ Animals and other organisms
☒	☐ Clinical data
☒	☐ Dual use research of concern
☒	☐ Plants

Methods

n/a	Involved in the study
☒	☐ ChIP-seq
☐	☒ Flow cytometry
☒	☐ MRI-based neuroimaging

Antibodies

Antibodies used	The detailed information about antibodies is provided in the reagent part.
Validation	The commercial antibodies used in this study were validated by the manufacturer. In our study, antibody-specific staining was compared to isotype and control samples.

Eukaryotic cell lines

Policy information about cell lines and Sex and Gender in Research

Cell line source(s)	RAW 264.7 was obtained from American Type Culture Collection (ATCC). iBMDM was donated by Prof. Haipeng Liu from Shanghai Pulmonary Hospital (Ma, M., Mol Cell 2023).
Authentication	The cell lines have been authenticated using STR profiling.
Mycoplasma contamination	The cell lines were routinely tested as mycoplasma-free before use.
Commonly misidentified lines (See ICLAC register)	No commonly misidentified cell lines were used in this manuscript.

Animals and other research organisms

Policy information about studies involving animals; ARRIVE guidelines recommended for reporting animal research, and Sex and Gender in Research

Laboratory animals	C57BL/6J mice (6-8 weeks old) were obtained from the Joint Ventures Sipper BK Experimental Animal Company (Shanghai, China). G3bp1f/f mice were constructed by Cyagen Biosciences Corporation (Suzhou, China). Ly6g-Cre mice and Siglecf-Cre mice were constructed by Shanghai Biomodel Organism Science & Technology Development Corporation (Shanghai, China). Lrp1 f/f mice (No. T064127) and Csf1r-Cre mice (No. T005640) were constructed by GemPharmatech (Nanjing, China). Lyz2-Cre mice (No. 004781) were obtained from The Jackson Laboratory.
Wild animals	The study did not involve wild animals.
Reporting on sex	Sex was not considered in study design.
Field-collected samples	There was no study involving samples collected from the field.
Ethics oversight	Animal experimentation was approved by the Committee on Ethics of Medicine, Second Military Medical University.

Note that full information on the approval of the study protocol must also be provided in the manuscript.

Plants

Seed stocks	*Report on the source of all seed stocks or other plant material used. If applicable, state the seed stock centre and catalogue number. If plant specimens were collected from the field, describe the collection location, date and sampling procedures.*
Novel plant genotypes	*Describe the methods by which all novel plant genotypes were produced. This includes those generated by transgenic approaches, gene editing, chemical/radiation-based mutagenesis and hybridization. For transgenic lines, describe the transformation method, the number of independent lines analyzed and the generation upon which experiments were performed. For gene-edited lines, describe the editor used, the endogenous sequence targeted for editing, the targeting guide RNA sequence (if applicable) and how the editor was applied.*
Authentication	*Describe any authentication procedures for each seed stock used or novel genotype generated. Describe any experiments used to assess the effect of a mutation and, where applicable, how potential secondary effects (e.g. second site T-DNA insertions, mosiacism, off-target gene editing) were examined.*

Flow Cytometry

Plots

Confirm that:

- [x] The axis labels state the marker and fluorochrome used (e.g. CD4-FITC).
- [x] The axis scales are clearly visible. Include numbers along axes only for bottom left plot of group (a 'group' is an analysis of identical markers).
- [x] All plots are contour plots with outliers or pseudocolor plots.
- [x] A numerical value for number of cells or percentage (with statistics) is provided.

Methodology

Sample preparation	Cells were isolated from mice nasal mucosa tissues, bone marrow and peritoneal cavity. Less than 1 million cells were washed with PBS, labeled in 100 µL PBS containing the relevant antibodies, and incubated at 4°C in the dark for 15 minutes. Samples were washed in 0.5 mL PBS twice before running.
Instrument	Fortessa flow cytometry (BD)
Software	FlowJo software (BD)
Cell population abundance	No cell sorting was performed.
Gating strategy	All samples are gated on single cells (Using FSC-A/FSC-H and SSC-A/SSC-H). Frequency of positive gates is determined using a biological control or an isotype control. Gating strategy is provided in the Supplementary Information.

- [x] Tick this box to confirm that a figure exemplifying the gating strategy is provided in the Supplementary Information.
