# Supplementary material for: Stress granule assembly impairs macrophage efferocytosis to aggravate allergic rhinitis in mice
Source: Nat Commun. 2025 Jul 1;16:5610. doi: 10.1038/s41467-025-60920-0 (PMC12218239; doi:10.1038/s41467-025-60920-0)
Supplement: Supplementary file 4 — Description of Additional Supplementary Files [file 41467_2025_60920_MOESM4_ESM.pdf]

Description of Additional Supplementary Files

Supplementary Movie 1

Live-video imaging of RAW 264.7 cells expressing G3BP1-GFP (green) followed by $NaAsO_2$ administration. The movie shows the recovery of fluorescence (green) after a pulse bleached 70%-80% of the fluorescence in 1 min. Scale bar, 25 μm.

Supplementary Movie 2

Live-video imaging of RAW 264.7 cells expressing G3BP1-GFP (green) followed by HDM administration. The movie shows the recovery of fluorescence (green) after a pulse bleached 70%-80% of the fluorescence in 1 min. Scale bar, 25 μm.

Supplementary Movie 3

Live-video imaging of NMMs expressing G3BP1-GFP (green) followed by HDM administration. The movie shows the recovery of fluorescence (green) after a pulse bleached 70%-80% of the fluorescence in 1 min. Scale bar, 25 μm.

Supplementary Movie 4

G3bp1$^{mac-/-}$ macrophages preferentially phagocytizes apoptotic Jurkat cells. HDM-administrated PKH26-labeled *G3bp1*$^{f/f}$ macrophages (Red) and PKH67-labeled *G3bp1*$^{mac-/-}$ macrophages (Green) were co-cultured with CellVue® Claret-labeled apoptotic Jurkat cells (Cyan). The phagocytosis of apoptotic Jurkat cells by macrophages was detected by time-lapse imaging using the Leica confocal microscope, showing that *G3bp1*$^{mac-/-}$ macrophages (green), but not *G3bp1*$^{f/f}$ macrophages (Red), preferentially phagocytizes apoptotic Jurkat cells (Cyan). Scale bar, 25 μm.
